# The Efficacy of Laser Therapy in Melasma Treatment: A Systematic Review and Meta‐Analysis

**DOI:** 10.1111/jocd.70602

**Published:** 2025-12-11

**Authors:** Masoomeh Chehrara, Anahita Tabavar, Masoumeh Roohaninasab, Paria Jafary, Arash Pour Mohammad, Samaneh Mozafarpoor, Ehsan Alavi Rad, Azadeh Goodarzi

**Affiliations:** ^1^ Iran University of Medical Science Tehran Iran; ^2^ Department of Dermatology, Hazrat Fatemeh Hospital, School of Medicine Iran University of Medical Sciences (IUMS) Tehran Iran; ^3^ School of Medicine Iran University of Medical Sciences Tehran Iran; ^4^ Skin Diseases and Leishmaniasis Research Center, Department of Dermatology Isfahan University of Medical Sciences Isfahan Iran; ^5^ Department of Radiology, School of Medicine Tehran University of Medical Sciences Tehran Iran

**Keywords:** Er:YAG, Erbium YAG, laser, laser therapy, melasma, meta‐analysis, QSNDY, Q‐switched Nd:YAG, super skin rejuvenation, systematic review

## Abstract

**Introduction:**

Laser treatment is one of the most common methods currently used to treat melasma. Here in, we reviewed RCTs with at least one laser therapy arm used in net form for treatment, so as to assess the use of laser therapy for melasma treatment with a degree of certainty.

**Methods:**

A systematic literature search was performed on the effectiveness of laser therapy for melasma treatment in the period from 2010 to 2024 using the keywords *melasma*, *laser*, and *efficacy* in the title. Finally, a meta‐analysis was conducted using the mean and standard deviation of the Melasma Area and Severity Index (MASI) variations before and after each session of laser therapy in different studies.

**Results:**

Sixteen RCTs on the efficacy, safety, and recurrence associated with laser therapy for melasma were eventually included and assessed. A total of 471 patients with a mean age of 35.3–46.4 years were examined. The 1064‐nm Q‐Switched Nd:YAG Laser (QSNd:YAG) was the most common laser investigated in the studies. There were 2–12 treatment sessions, with 1–4‐week intervals, and the patients' follow‐up period was 2–6 months. The use of SSR (Super Skin Rejuvenation, 540 nm), low‐energy QSNDY, and pixel‐Er:YAG (Erbium YAG) for five sessions at 3‐week intervals showed a very significant decrease in the modified MASI (mMASI) score (*p* < 0.001). Pixel‐Er:YAG showed the maximum mMASI reduction and homogeneity (*p* > 0.001). Epidermal melasma had the best results with SSR and PQSND (Y Pixel Q‐Switched Nd:YAG Laser) (*p* < 0.001).

**Conclusion:**

Among the reviewed RCTs, the largest weight was assigned to a 2014 study that examined 40 patients (4.79% weight). The standardized variation of the mean MASI was negative in all studies, except for a 2011 study, which had the lowest weight among the studies (2.84%), indicating the mitigation of symptoms following laser therapy. A meta‐analysis of 16 research studies (26 treatment arms) was performed to determine the efficacy of laser therapy for melasma. Treatment resulted in a significant improvement in MASI scores, with a pooled standardized mean difference of 0.88 (95% CI: 0.65–1.11, *p* < 0.00001). Due to moderate‐to‐high heterogeneity (*I* ^2^ = 68.4%), a random‐effects model was employed. Overall, the findings indicate that for the majority of patients, laser therapy reduces pigmentation and lesion severity.

## 1 Introduction

Melasma is a common and chronic condition that primarily affects those with darker skin tones. It appears as hyperpigmented patches and macules on sun‐exposed areas, particularly the face [1]. Although the precise cause of melasma formation is unknown at this time, a number of factors, such as UV radiation, hormonal shifts in the estrogen or progesterone pathways, genetic predisposition, and/or inflammation, are known to have a role in the condition's development [2]. The quality of life is severely compromised by melasma [2]. It is also not readily treated, although there are a number of options available, including chemical peels, topical medications, and other comparable interventions [3]. Because cutaneous melanosomes are difficult to cure, topical medications and superficial peels are rarely effective [4]. Because of their exact selection, deep penetration, and capacity to preserve the epidermis, lasers are essential in the treatment of various disorders [5]. Key elements facilitating the efficient targeting of skin pigments include laser therapy's ultra‐short pulse width (in nanoseconds), customizable spot size, and capacity to precisely target melanosomes in melanocytes, keratinocytes, and melanophages [6]. Although lasers have transformed the treatment of skin conditions, there is ongoing debate on their applicability in the management of melasma and post‐inflammatory hyperpigmentation (PIH) [7]. There isn't a true gold standard treatment for melasma, despite the fact that it's a very frequent skin problem that responds poorly to therapies as its first line of treatment [8].

Thus far, a great deal of study has been done on effective, safe, and long‐lasting melasma treatment techniques. Although laser therapy has become a popular treatment option in recent years, its effectiveness in treating melasma has not been well studied. Thus, by analyzing randomized controlled trials (RCT) with at least one laser therapy arm, this study sought to investigate the effectiveness of laser therapy.

## 2 Methods

### 2.1 Research Design and Study Population

This was a systematic review and meta‐analysis. The population consisted of studies carried out on the efficacy of laser therapy for melasma from 2010 to 2024.

### 2.2 Sampling

Relevant studies were collected using PICO(s) search strategy in the Cochrane Library, PubMed/MEDLINE, Evidence, gray literature (Proquest), OVID, Scopus, WoS, and Embase databases. Articles published from January 1, 2010, to August 1, 2024, with language restrictions (only English) were extracted using different combinations of the following keywords: Safety, efficacy, recurrence, laser and melasma, (Melasma [Title/Abstract) OR [Title/Abstract], Solar Lentigines) AND (laser [Title/Abstract) OR (Q‐switched [Abstract/Title), and their Persian equivalents (*melasma, laser* and *efficacy*). After searching and selecting the articles, their full text was qualitatively evaluated using the PRISMA checklist, and they were included in the analysis. A manual search was also performed in case the database did not follow the search syntax.

### 2.3 Data Collection Tools

Data were collected by searching the Cochrane Library, PubMed/MEDLINE, Evidence, gray literature (Proquest), OVID, Scopus, WoS, and Embase databases. Finally, the articles were scored using the EBL checklist.

### 2.4 Procedure

This systematic review was first approved by the Research Center, and the University's Medical Research Ethics Committee. After collecting the documents and articles on the efficacy and safety of laser therapy for melasma treatment, their details and abstracts were imported to Endnote, and duplicates were removed with this software as well as by re‐reading the titles. Next, after the titles were reviewed, studies irrelevant to the research objectives were excluded; for the remaining studies, the abstract and full text were reviewed to ensure that they were relevant to the objectives of the present study, and any irrelevant studies were again removed. Searches were limited to studies conducted only on humans. Finally, the studies published in English were included in this review study. Only RCT studies that had a net laser therapy arm were included; this treatment arm could not be combined with other treatments, and the net effect of laser therapy had to have been assessed. Variables including the severity of melasma, type of laser, number of laser therapy sessions, interval between laser therapy sessions, duration of follow‐up, the efficacy of treatment, the safety of treatment, and recurrence were included in the table of variables. After filling out the EBL checklist, the studies that were biased in sample selection, sampling method, and type of analysis were scored according to the checklist, and those that scored below 75% were excluded. Moreover, the included articles had to have reported the mean of the intended outcomes and standard deviation or confidence interval or *p* value.

### 2.5 Data Analysis Method

First, the consistency and homogeneity between the studies were calculated using the relevant charts and the *I* ^2^ index. After determining the articles to be included, relevant charts such as the funnel plot were used to check the publication bias, which showed no need for adjustment. In the case of homogeneity, the fixed‐effects analysis is used in the meta‐analysis; otherwise, random effects analysis is utilized. In general, a forest plot is used to combine the effect sizes. The significance level of this study was set to 0.05. The analyses were performed in STATA.

### 2.6 Ethical Considerations

The researchers were committed and adhered to the principles of the Declaration of Helsinki and the Ethics Committee of dermatology.

## 3 Results

**FIGURE 1:**
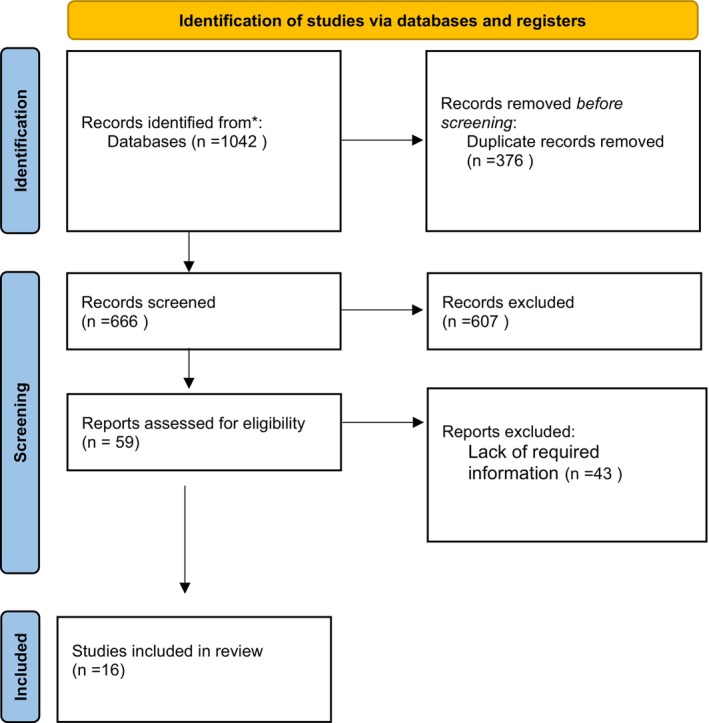
PRISMA chart.

After the initial search using the specified keywords, 1042 articles were extracted from the databases. Their titles were imported to Endnote and 376 duplicates were removed. Then, by limiting the articles to the 2010–2024 time frame, 182 other articles were removed. For the remaining 484, the titles were examined, and 425 other articles were excluded and 59 remained. Finally, by carefully examining their full text, 42 other articles were excluded as they lacked the required information, and finally, 16 articles were included in the final review (Figure 1).

Sixteen RCTs examining the efficacy, safety, and recurrence associated with laser therapy for melasma were included and reviewed in this systematic review and meta‐analysis. The articles had been published between 2011 and 2024 and examined 471 patients in total. The patients' mean age was 35.3–46.4 years. In 16 articles, 97.5% of the patients were women (Tables 1 and 2). The 1064‐nm Q‐Switch Nd:YAG laser was the most common laser investigated in the studies. There were 2–12 treatment sessions, with 1–4‐week intervals. The follow‐up period was 2–6 months.

**TABLE 1:** Data extracted from the 16 studies (1).

Authors	Year of publication	No. of patients	Mean age	Sex (%)	Type of laser
Hei et al. [9]	2013	26	45	100	1064 nm QSNY
		26	45	100	1064 nm QSNY+NFP
Yun et al. [10]	2015	26	41.9	100	Conventional IPL
		26	41.9	100	Fractional IPL
Kroon et al. [11]	2011	10	35.3	100	Nonablative 1550‐nm fractional laser
Park et al. [12]	2011	16	43.94	100	1064 nm QSNd:YAG
Shin et al. [13]	2012	21	43.2	100	Low‐fluence 1064‐nm QSNd:YAG
Jalaly et al. [14]	2014	40	42.5	100	Low power fractional CO2
		40	42.5	100	1064 nm QSNd:YAG
Yun et al. [15]	2014	12	42.6	100	IPL‐F + QSNd:YAG
		12	43.4	100	IPL‐F
Dev et al. [16]	2020	28	37	100	1064 nm QSNd:YAG
Ustuner et al. [17]	2017	16	37.69	100	1064 nm QSNd:YAG
Lee et al. [18]	2014	26	42.08	100	1064 nm QSNYL
Wang et al. [19]	2020	11	46.4	100	Picosecond alexandrite laser with a diffractive lens array
	2020	9	40.4	100	Picosecond alexandrite laser with a diffractive lens array
Garg et al. [20]	2019	20	(22–46)	80	540 nm SSR
		20	(22–46)	80	1064 nm QSNd:YAG
		20	(22–46)	80	2940 nm Er:YAG
Botsali et al. [21]	2022	34	40.4	100	Fractional erbium:YAG laser
Han et al. [22]	2024	25	40.56	100	730‐nm picosecond laser
Liang et al. [23]	2023	20	37.8	100	1064 nm picosecond Nd:YAG laser
		20	39.5	100	755 nm picosecond alexandrite laser
Beyzaee [24]	2021	30	39.97	100	Q‐Switched Nd:YAG Laser
		30	39.97	100	Fractional CO_2_ laser

**TABLE 2:** Data extracted from the 16 studies (2).

Authors	Type of laser	No. of treatment sessions	Interval between sessions	Follow‐up duration	Device/brand	setting	Safety	Recurrence	Percentage of patients' satisfaction
Kim et al. [9]	1064 nm QSNY	10	2 weeks	12 weeks	MedLite C3TM; Hoya Conbio Inc., Fremont, CA, USA	6 mm spot size, energy fluence 1.2–1.4 J/cm2 at 10 Hz	Erythema, temporary burns, mild edema	—	65.4
	1064 nm QSNY+NFP	10	2 weeks	12 weeks	MedLite C3TM; Hoya Conbio Inc., Fremont, CA, USA + MOSAICTM, Lutronic Corporation, Seoul, South Korea	6 mm spot size, energy fluence 1.2–1.4 J/cm^2^ at 10 Hz + dynamic mode, pulse energy 6–8 mJ/microthermal zone (MTZ); MTZ diameter: 150 μm; total density: 300 MTZs/cm^2^	Erythema, temporary burns, mild edema	—	73.1
Yun et al. [10]	Conventional IPL	6	1 week	14 weeks	Ellipse Flex (Danish Dermatologic Development, Hoersholm, Denmark)	530‐nm to 750‐nm filter	PIH in one patient	—	64
	Fractional IPL	6	1 week	14 weeks	EclatST (Union Medical, Seoul, South Korea)	550‐nm to 850‐nm filter, 40–200 fractionated subpulses within a microsecond time frame	Mild increase in pigmentation in one patient	—	64
Kroon et al. [11]	Non ablative 1550‐nm fractional laser	4	2 weeks	6 months	Fraxel Re:store laser, Reliant Technologies Inc., Mountain View, CA	final density of 2000 to 2500 microscopic treatment zones/cm^2^, and 4 passes, one direction and 4 perpendicularly	Sunburn‐like erythema in 75% of the patients	50%	62.5
Park et al. [12]	1064 nm QSNd:YAG	6	1 week	5 months	MedLite C6; Hoya ConBio, Fremont, CA, USA	6‐mm spot size, collimated homogenous flat‐top beam profile, energy fluence 2.0–2.3 J/cm^2^ at 10 Hz	Erythema, temporary burns, mild edema	—	38
Shin et al. [13]	Low‐fluence 1064‐nm QSNd:YAG	2	4 weeks	2 months	VRM IV Spectra, Lutronic Corporation, Goyang, Korea	2.0 J/cm^2^ and a spot size of 7 mm	Transient erythema	—	57.1
Jalaly et al. [14]	Low power fractional CO_2_	5	3 weeks	2 months	MixelÒ; Hironic Co. Ltd., Korea	Power of 1 Wand density of 0.7	Erythema, burning sensation, and transient edema	—	82.5
	1064 nm QSNd:YAG	5	3 weeks	2 months	SpectraÒ; Lutronic Inc., Korea	1.5–2 J/cm^2^ with 7 mm spot sizes with five passes	Sunburn‐line erythema, transient edema	—	65
Yun et al. [15]	IPL‐F + QSNd:YAG	6	2 weeks	2 months	EclatST; Union Medical, Seoul, Korea+VRMIII; Lutronic, Seoul, Korea	IPL‐F ranged from 13 to 15 J/cm2 for 4–6 passes+fluence ranging from 1.5 to 2.0 J/cm^2^, a spot size of 6 mm, a pulse duration of 5 to 10 ns, and a frequency of 10 Hz	Transient first‐degree burns in one patient, skin dryness in three patients	—	41.7
	IPL‐F	6	2 weeks	2 months	EclatST; Union Medical, Seoul, Korea	IPL‐F ranged from 13 to 15 J/cm^2^ for 4–6 passes	No serious side effects	—	16.7
Dev et al. [16]	1064 nm QSNd:YAG	12	1 week	3 months	Med‐lite C6 Hoya Con Bio Inc., Fremont, CA, USA	Fluence: 1.5 J/cm^2^; spot size: 6 mm; frequency: 10 Hz; 10 passes or until pigment lightening and hair whitening observed	Acute transient hives in one patient	—	86
Ustuneret al. [17]	1064 nm QSNd:YAG	4	4 weeks	12 weeks	Not mentioned	First pass: 8‐mm spot, 2.0 J/cm^2^; second pass: 6‐mm spot, 3.5 J/cm^2^; final pass: 4‐mm spot, 3.2 J/cm^2^, followed by multiple passes over lesions until mild erythema and swelling appeared	Hypo‐ or hyperpigmentation in two patients (31.3%)	43.8%	14.3
Lee et al. [18]	1064 nm QSNYL	10	2 weeks	20 weeks	Cosjet TR, Won Technology, Korea	7‐mm spot size, collimated homogenous flat‐top beam profile, energy fluence 1.0–1.7 J/cm^2^ at 10 Hz. Two passes	Mild pain and erythema	—	80.7
Wang et al. [19]	Picosecond alexandrite laser with a diffractive lens array	5	4 weeks	20 weeks	PicoSure, Cynosure, MA, USA + FOCUS Lens Array	755‐nm picosecond laser with DLA; fluence: 0.4 J/cm^2^; spot size: 8 mm; pulse duration: 750 ps; two passes. DLA delivered ~2.8 J/cm^2^ to 10% of tissue (microbeams) and ~0.13 J/cm^2^ to 90% (background)	27.3% erythema, 18.2% PIH, 27.3% focal desquamation	0	38
	Picosecond alexandrite laser with a diffractive lens array	3	4 weeks	20 weeks	PicoSure, Cynosure, MA, USA + FOCUS Lens Array	755‐nm picosecond laser with DLA; fluence: 0.4 J/cm^2^; spot size: 8 mm; pulse duration: 750 ps; two passes. DLA delivered ~2.8 J/cm^2^ to 10% of tissue (microbeams) and ~0.13 J/cm^2^ to 90% (background)	22.2% erythema, 11.1% focal desquamation	0	53
Garg et al. [20]	540 nm SSR	5	3 weeks	6 months	Harmony‐XL; Alma Lasers Ltd., Caesarea, Israel	8–9 J/cm^2^, one pass of double shots	Transient skin dryness	—	90
	1064 nm QSNd:YAG	5	3 weeks	6 months	Harmony‐XL; Alma Lasers Ltd., Caesarea, Israel	Pixel tip, frequency 4 Hz, low fluence of 1.2 J/cm^2^, 4 passes	PIH in three patients	—	80
	2940 nm Er:YAG	5	3 weeks	6 months	Harmony‐XL; Alma Lasers Ltd., Caesarea, Israel	Pixel tip, 1100–1200 mJ/p, long pulse, stack mode, 4–5 passes	PIH in two patients	—	85
Botsali et al. [21]	Fractional erbium:YAG laser	4	2 weeks	3 weeks	Fotona Dynamis, XS, Slovenia, Ljubljana	300 μs pulse, 1.2 J/cm^2^, 5 mm spot applied to full face. *Second pass*: Same settings applied to affected areas. Fluence was later adjusted based on Fitzpatrick skin type and patient response, reaching 3–6 J/cm^2^ in subsequent sessions	Erythema	—	73.2
Han et al. [22]	730‐nm picosecond laser	3–5	4–13 weeks	4–13 weeks	Picoway; Candela Medical, Marlborough, MA	Pulse duration: 246 ps; spot sizes: 2, 3, and 4 mm; energy range: 0.6–1.0 J/cm^2^ for 4‐mm spot size	Mild erythema	—	36
Liang et al. [23]	1064 nm picosecond Nd:YAG laser	3	4 weeks	24 weeks	Picoway; Candela, Massachusetts, United States	Non‐fractional, Pulse duration:450 ps, spot size:7 mm, fluence:0.75–0.90j/cm^2^, repitation: 8, two passes, Average pulses per treatment: 4500, endpoint: mild erythema	Transient post laser hyperpigmentation	5%	35.9
	755 nm picosecond alexandrite laser	3	4 weeks	24 weeks	Picosure; Cynosure, MA, United States	Non‐fractional, Pulse duration:750 ps, spot size: 6–8 mm, fluence:0.40–0.71/cm^2^, repitation: 8, two passes, Average pulses per treatment: 4100, endpoint: mild erythema	Transient post laser hyperpigmentation	10%	25.5
Beyzaee et al. [24]	Q‐Switched Nd:YAG Laser	6	2 weeks	2 weeks	JEISYS company, Seoul, Korea	Pulse energy: 750 mJ, fluence: 1.50 J/cm^2^, spot size: 4 mm *×* 4 mm, hand piece: fractional	Transient erythema and discomfort	—	61.7
	Fractional CO_2_ laser	6	2 weeks	2 weeks	LUTRONIC company, Gyeonggi‐do, Korea	Power: 30 w, pulse energy: 30 mJ, tip type: 300, pulse rate: 100/cm^2^	Transient erythema and discomfort	—	73.25

### 3.1 Meta‐Analysis Results

To investigate the effect of laser therapy in melasma treatment, standardized mean differences (SMDs) in MASI scores before and after treatment were meta‐analyzed. Due to substantial between‐study heterogeneity (*I* ^2^ = 68.4%, *τ* ^2^ = 0.2354, *χ* ^2^ = 74.43, *p* < 0.0001), a random‐effects model using Restricted Maximum Likelihood (REML) estimation was adopted to account for variability.

Each study was weighted based on its precision, and the final pooled effect was calculated across 16 studies (26 treatment arms in total). The standardized mean difference was estimated at 0.88 [95% CI: 0.65–1.11] (*p* < 0.00001), indicating a statistically and clinically significant improvement in melasma following laser therapy.

**FIGURE 2:**
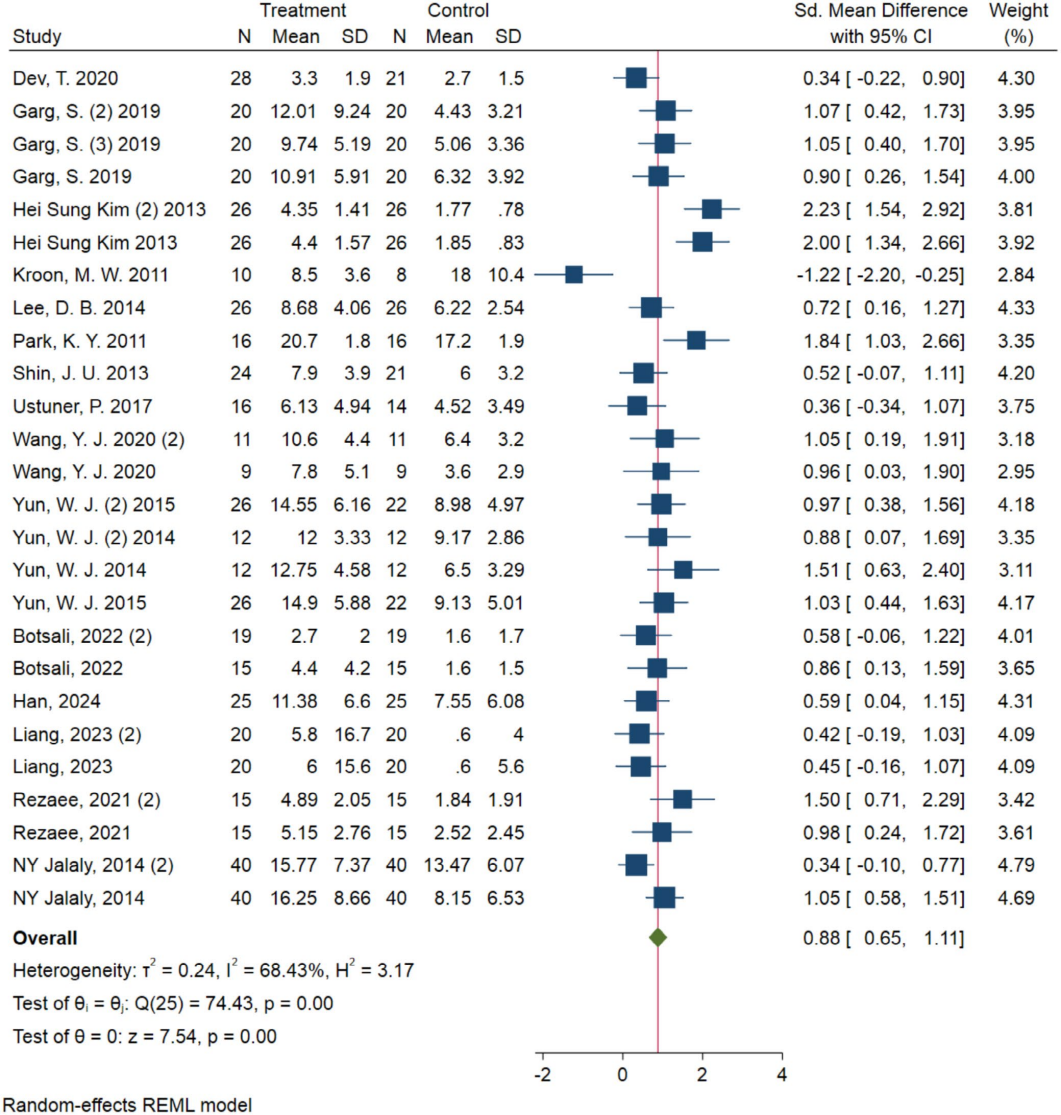
Meta‐analysis of the data from the 16 studies according to MASI scores before and after laser therapy for melasma treatment.

Notably, this effect size corresponds to a moderate‐to‐large clinical improvement (> 0.8), suggesting that most patients experience a meaningful reduction in pigmentation and lesion severity after undergoing laser‐based treatment (Figure 2).

## 4 Discussion

Women with skin phenotypes IV–VI are the most common to suffer from melasma, a common acquired hypermelanosis [25]. Although the exact process underlying the formation of melasma remains unclear, recent research indicates that it is a complex disorder involving disruptions to the pigment homeostasis pathways in the dermis, extracellular matrix, and epidermis [26]. The net effect of these modifications is an increase in the pigment production pathways relative to the overall pigment removal pathways. Melanosomes consequently build up in the DEJ, papillary dermis, or deeper [27]. Although lasers have transformed the treatment of skin conditions, there is ongoing debate on their use in the therapy of melasma and PIH.

This overview of numerous studies highlights how crucial laser settings are to achieving optimal results with the fewest possible adverse effects. When treating melasma, selecting the appropriate laser and settings is essential. In treating melasma, laser therapies have demonstrated encouraging outcomes, particularly for patients with darker skin tones. Melasma can be treated using a variety of laser modalities, including Q‐switch, Erbium:YAG, and ablative and nonablative fractional lasers [28].

### 4.1 Q‐Switch Nd:YAG(QSNY) in Combination With Other Lasers

The QSNY laser is the most commonly utilized laser for melasma; however, in a number of papers, this laser has been used in conjunction with other lasers to determine whether or not its efficacy has risen. Kim et al.'s [9] experiment examined the efficacy of treating women with melasma (Fitzpatrick skin phenotypes III–V) with low‐fluence 1064 QSNY in combination with nonablative fractional 1550 nm (NFP) laser against low‐fluence 1064 QSNY treatment alone. A total of 26 eligible individuals had 10 sessions of 1064 QSNY laser therapy, plus five more NFP laser sessions for one side of their face. At baseline, 4 weeks, and 12 weeks after therapy, the modified Melasma Area and Severity Index (mMASI) was utilized to evaluate the severity of melasma. Although both treatments exhibited improvement over time, the results did not reveal a significant difference in mMASI scores between the two treatment sides at baseline or at follow‐ups. There was no discernible difference in the patient‐reported results between the two groups at the 12‐week follow‐up; 38.4% of patients in the combination treatment group and 42.3% in the 1064 QSNY group reported virtually whole or distinct clearance. Patients thought both treatment plans were mostly effective.

Forty healthy female patients over the age of 18 who had Fitzpatrick skin phenotypes II–IV and symmetrical melasma participated in a study by Jalay et al. [14]. Five low‐fluence Q‐switch 1064 nm Nd:YAG laser passes were made on one side of each patient's face, and one low‐power fractional CO_2_ laser pass was made on the other. Five sessions of treatment were given every 3 weeks, and then there was a 2‐month monitoring period. Standardized pictures were taken for assessment, and the Melanin Index (MI) was calculated to gauge the improvement in hyperpigmentation. The modified Melanin Area and Severity Index (mMASI) was utilized by a dermatologist who was blinded to evaluate pigmentation. MI and mMASI ratings did not differ at the first visit between the two treatment groups. Both therapies, however, demonstrated a significant decrease in MI and mMASI scores at the 2‐month follow‐up. While there were no statistically significant variations in MI scores, the fractional CO_2_ laser side showed a higher drop in mMASI scores when compared to the Q‐switch laser. Patients with epidermal and dermal melasma showed a greater reduction in MI scores when treated with the fractional CO_2_ laser.

Sixty patients with recalcitrant melasma were split into three therapy groups in a research study by Garg et al. [20]. Group B experienced pixel Q‐switched Nd: YAG 1064 nm (PQSNDY), Group C received ablative Pixel Er: YAG (2940 nm) laser, and Group A received Super Skin Rejuvenation (SSR) 540 nm. Every patient received five sessions of treatment spaced 3 weeks apart. Assessments were done at each visit and 6 months after the last session. Results showed that all patients in the SSR and PQSNDY groups experienced fading. Among the 20 participants in the Pixel Er: YAG group, only 12 had pigmentation. The mMASI showed the largest overall reduction in the Pixel Er: YAG group, but the difference was not statistically significant when compared to the other groups. Every group showed a notable improvement in mMASI. All forms of melasma responded well to SSR, especially in cases of epidermal melasma; PQSNDY produced comparable outcomes. For dermal and mixed melasma, the Pixel Er: YAG group showed significant outcomes, but not for epidermal melasma. An additional advantage mentioned for all patients was facial rejuvenation.

Beyzaee et al. [24] studied the efficiency of a fractional CO_2_ laser and a Q‐Switched Nd:YAG (QSNY) laser, both paired with oral tranexamic acid (TA), in treating refractory melasma. Thirty patients underwent fractional CO_2_ laser on one side of the face and QSNY laser on the other, with TA 250 mg twice daily for six sessions. The fractional CO_2_ laser group outperformed the QSNY group in terms of patient and physician assessments (PtGA and PGA) and Melasma Area and Severity Index (MASI) scores. Although both treatments were effective over time, the fractional CO_2_ laser outperformed them. Common side effects such as erythema and pain were modest and subsided within 24 h. The study found that fractional CO_2_ laser combination with oral TA is an efficient and well‐tolerated treatment for refractory melasma.

### 4.2 Q‐Switch Nd:YAG(QSNY) in Combination With Oral or Topical Medication

Before the development of lasers, the first line of treatment for melasma was oral or topical medications. With the introduction of lasers, these treatments were moved to new areas, but it has long been unclear if the combined use of medication and lasers can improve results over the use of either one alone.

In order to test the efficacy of a combination treatment of low‐fluence 1064 QNYL laser and glycolic acid (GA) peel against low‐fluence 1064 QNYL treatment alone in patients with mixed type melasma, Park et al. [12] conducted a single‐center, split‐face, randomized, observer‐blinded trial. The combined therapy or the laser treatment alone was randomly assigned to each participant's face, and each side had six weekly sessions of laser therapy. Following the laser treatment, three sessions of 30% GA were administered to one side at intervals of 2 weeks. The study was completed by all 16 individuals, and at each visit, the MI was measured. The side receiving combination therapy had a noteworthy 32.6% improvement in pigmentation, whereas the side receiving QNYL alone demonstrated a 22.0% improvement. Additionally, there was a considerable drop in the mean mMASI, with a reduction of 37.4% for the combined therapy and 16.7% for the laser‐only side. Independent dermatologists observed a more than 50% improvement in 69% of patients receiving combination therapy during the 5‐month follow‐up, compared to 31% of patients receiving only laser treatment. Furthermore, compared to 38% in the laser‐only group, 75% of patients in the combination therapy group assessed their outcomes as satisfactory or excellent. The majority of patients also mentioned advantages including smoother, lighter skin.

Shin et al. [13] begin 48 healthy Korean women with melasma, aged 18–55, who were included in the study. They were divided into two groups at random and given oral TA and low‐fluence QSNY laser treatment, or low‐fluence QSNY laser treatment alone. While the laser‐only group followed the same laser treatment schedule, the combination group received two laser sessions spaced 4 weeks apart in addition to taking 750 mg of oral TA daily for 8 weeks. The mMASI and assessments by two blinded dermatologists based on baseline and 2‐month post‐treatment photos were used to gauge the degree of patient improvement.

Dev et al.'s [16] split‐face randomized controlled study consisted of two stages: a 12‐week treatment phase and 3 months of monthly follow‐ups. Patients were randomized into two groups: group B received a Modified Kligman's triple combination (TC) treatment, whereas group A received weekly QSNYL. 38 of the 93 patients who were screened, 45 who were recruited, and the study was completed. After a 12‐week period, group A's pigmentation improved by 17.3%, while group B's improved by 20.9%. Treatment outcomes did not significantly differ between the two groups, and the type of melasma had no bearing on the outcome. Following treatment, the mMASI scores of both groups exhibited significant declines, with the combination group showing a higher drop in scores than the laser‐only group. On the other hand, neither group's MI changed significantly.

Ustuner et al.'s [17] double‐blind, randomized controlled study, which included 16 Turkish melasma patients, assessed the results of laser treatment in addition to microneedling. There were two groups of patients: Group B only received the laser therapy; Group A received three passes of the QS‐Nd:YAG laser in addition to vitamin C‐based microneedling. MelasQoL‐TR was used to measure patient satisfaction, and MASI scores were used to measure clinical responses. Group A's MASI ratings were significantly higher at the beginning, but by months one through four, they were significantly lower than Group B's, indicating superior clinical outcomes.

52 female patients participated in Lee et al.'s study [18], where they were divided into two groups at random. Group A had a placebo and a 1064‐nm QSNYL laser therapy, while Group B got the same laser treatment with Jessner's peel. The Melasma Area and Severity Index (MASI) and Physician's Global Assessment (PGA) were used to assess the treatment groups' responses over a 20‐week period after they received 10 sessions of treatment spaced 2 weeks apart. The findings demonstrated that the mean MASI scores of both groups significantly decreased from baseline, with Group B experiencing a drop of 32.6% and Group A seeing a reduction of 28.3%. Group B showed a statistically significant drop in MASI scores at 8 weeks, but by 20 weeks, this difference was no longer significant. While not statistically significant, a greater proportion of patients in Group B (80%) than in Group A (73%) reported greater than average improvement. In both groups, patient satisfaction scores were comparable.

### 4.3 Intense Pulse Light Lasers

Yun et al. [15] conducted a study on 30 healthy Korean women with melasma who were aged 28–59. The patients were randomly assigned to receive either standard IPL or new fractionated intense pulsed light (IPL) treatment on each cheek. The traditional IPL group received treatments every 2 weeks for a total of three sessions, whereas the fractionated IPL group had weekly treatments for 6 weeks. The treatment strategy was standardized for the trial and was not modified based on individual responses. The participants were followed up with 1 and 2 months after the therapy. Two separate investigators examined the primary outcome, which was a decrease in MASI scores. Between the two therapy groups, no statistically significant differences were discovered. Nonetheless, the fractionated IPL regimen showed that it was not inferior to the standard IPL, and a larger drop in MASI scores was shown by the confidence interval. Remarkably, on the fourth visit, MASI scores on the traditional IPL side had recovered, whilst scores on the fractionated IPL side had been steadily declining. For all therapies, patient satisfaction scores were comparable, with more than 50% of respondents indicating excellent or good results. Following treatment, histological evaluations of biopsies obtained from the fractionated IPL‐treated side revealed a reduction in melanin pigment. Overall, the fractionated IPL demonstrated a greater positive trend in MASI score reduction over time, despite the effectiveness of both treatments.

In a different clinical trial conducted by Yun et al. [10], participants with melasma (Fitzpatrick skin phenotypes III or IV) who were in good health and aged 18–55 were randomized to receive either a combination therapy group or IPL‐only treatment. Six bimonthly IPL‐F and LF‐QS‐Nd:YAG laser treatments were administered to the combination group, while six IPL‐only sessions of IPL‐F were administered to the IPL‐only group. Standardized digital pictures were acquired for the baseline and 1‐ and 2‐month post‐treatment evaluations by two blinded dermatologists. The partial melasma area and severity index (MASI) score was the main outcome that was measured. The combo group outperformed the IPL‐only group, which had a 15% drop in MASI score, after 1 month, with a 47% reduction compared to baseline. The combination group continued to demonstrate a significant drop at 2 months post‐treatment, while the IPL‐only group displayed a 24% reduction from baseline. Over the course of the treatment, both groups saw a drop in their erythema index (EI) and MI. However, by the end of the treatment, the combined group had reduced its MI by 20.1%, while the IPL‐only group had only reduced its MI by 14.6%. The MI reduction in the IPL‐only group did not, however, continue during follow‐up.

### 4.4 Nonablative 1550‐Nm Fractional Laser Therapy

Using a sealed envelope randomization procedure, participants in Kroon et al.'s [11] trial were randomized to either a laser therapy group or a triple topical therapy group (hydroquinone, tretinoin, and triamcinolone). The course of therapy lasted from November to December, with follow‐up appointments planned for 3, 3, and 6 months after the end of the course of treatment. At the 3‐week follow‐up, the Physician Global Assessment measured hyperpigmentation in both groups, and improvements were noted; nevertheless, there were no statistically significant differences in terms of improvement or adverse effects between the groups. On the other hand, the laser group indicated far greater treatment satisfaction and recommendations.

### 4.5 Erbium:YAG Laser

This study, which was designed by Botsali et al. [21], looked at fractional erbium:YAG laser‐assisted delivery (LAD) of 5% TA, with or without oral TA, in intractable melasma patients. Two groups had four biweekly laser sessions: Group 1 received oral TA and LAD, and Group 2 received only LAD. Both groups experienced substantial mMASI score decreases (*p* = 0.001; *p* = 0.022), with Group 1 showing a higher improvement (64.7%) than Group 2 (41.8%) (*p* = 0.027). The study concludes that fractional erbium:YAG LAD is successful for melasma treatment, with oral TA improving outcomes.

### 4.6 Picosecond Alexandrite Laser

In Wang et al.'s [19] study, three groups of 29 female patients were randomized at random to assess various melasma treatments. Groups A1 and A2 underwent 4‐week intervals of three and five picosecond alexandrite laser treatments, respectively, whilst group B used a topical triple combination cream (TCC) every day for 8 weeks. The Melasma Area and Severity Index (MASI) was used to evaluate each group, and follow‐ups were conducted at weeks 12 and 20. All groups' MASI scores significantly improved, with group A1 showing the largest improvement (53%) and groups B and A2 showing the second and third largest improvements (50%) and 38%, respectively. For the laser groups, VISIA imaging showed improvements in skin texture and pigmentation.

Study of Liang et al. [23] investigated the efficacy and safety of non‐fractional picosecond Nd:YAG laser (PSNYL), picosecond alexandrite laser (PSAL), and 2% hydroquinone (HQ) in 60 individuals with Fitzpatrick skin phenotypes III‐IV. Over 24 weeks, all groups experienced significant MASI score reductions. PSNYL improved the most, outperforming PSAL (*p* = 0.016) and HQ (*p* = 0.018), although PSAL and HQ had similar outcomes. PSNYL provided the highest level of patient satisfaction. Recurrence occurred in 6.8% of instances, with temporary side effects. PSNYL was the most effective, making picosecond lasers a potential option for melasma treatment.

The 730‐nm picosecond laser has shown promise for treating melasma, although its efficacy and safety are still being investigated. This retrospective study, which was designed by Han et al. [22] examined 25 Chinese patients (Fitzpatrick skin phenotypes II‐IV) who had undergone laser treatment. After an average of 3.56 sessions, MASI scores reduced by 33.7% over 8.48 weeks (*p* < 0.001), with different levels of improvement. No occurrences of hyperpigmentation or hypopigmentation were found. The study found that a low‐fluence 730‐nm picosecond laser is a safe and effective treatment for melasma in Chinese patients.

### 4.7 Side Effects

Regarding the safety of employing lasers to treat melasma, Yun's study [15] found that one patient out of 26 receiving conventional IPL experienced PIH. 18.2% of the patients in Wang's [19], and Garg's [20] study (looking at Er:YAG and QSNd:YAG lasers) showed two and three patients, respectively, to have PIH. In addition to these instances, Yun's study [15], which examined the combined effects of IPL‐F + QSNd:YAG laser, involved the temporary first‐degree burns of one patient. One patient experienced brief acute hives in Dev T.'s research [16]. Apart from these instances, the majority of adverse effects noted in the studies were minor, with temporary erythema being the most common. The recurrence rate was estimated at 50% in Kroon's [11] study with a 6‐month follow‐up, and reported to be 43.8% after a 12‐week follow‐up in Ustuner's trial. After a 20‐week follow‐up, Wang's study [19]did not document any cases of recurrence. Statistics regarding the condition's recurrence were not provided by the other studies [19, 26].

The current meta‐analysis's findings are consistent with previous studies evaluating the effectiveness of laser therapy for melasma. Nine RCTs comprising 346 patients were analyzed in the study by Yanan Zhang et al. [29], and the mean fluctuation of mMASI scores following laser therapy was −1.57 with 95% CI (−0.05, −3.08) and I2 = 19%. The study's findings presented the use of laser as a means of reducing the size and intensity of melasma lesions [29]. The standardized differences in the mean MASI before and after laser therapy were investigated in this meta‐analysis using the random‐effects model. The resulting effect size was *p* < 0.00001 and had a 95% confidence interval of −0.60, −1.22. Thus, after merging the data from the 12 RCTs that were looked at, the efficacy of laser therapy for the treatment of melasma was determined to be significant in this meta‐analysis based on the standardized variations in MASI.

Dihui Lai et al.'s [7] study from 2022 included 22 RCTs involving 694 patients to compare the effectiveness of various laser kinds for treating melasma. The greatest mean alterations in MASI scores were obtained with the fractional ablative CO2 laser (−9.36 with 95% CI: −6.21, −12.51). This result, along with the 82.5% patient satisfaction rate with this kind of laser, makes the laser type noteworthy, and more research on it should be done in the future [28].

The safety of laser use was assessed by examining the side effects occurring after use; only Five studies [7, 15, 17, 27, 29] noted severe adverse effects, including temporary first‐degree burns, hyperpigmentation, and hives. Just three of the studies [17, 27, 30] included the percentage of recurrence, which ranged from 0% to 50%. After combining the study data based on sample size, the estimated level of patient satisfaction with this treatment approach was 66.2%.

The standardized differences in the mean MASI before and after laser therapy were examined in the current meta‐analysis using the random‐effects model. The study conducted by Jalaly et al. [14] on 40 patients had the highest weight (4.69% weight).

With the exception of Kroon et al.'s study [11], which had the lowest weight among the studies (4.2%), all of the investigations exhibited standardized variations in the mean MASI that were negative, indicating a decrease in patients' symptoms after laser therapy. At *p* < 0.00001, the computed final effect was −0.91 with a 95% confidence interval (−0.60, −1.22). Therefore, based on the standardized variations in the MASI score, the effect of laser therapy for melasma treatment was determined to be significant in the current meta‐analysis. After merging the data from the 12 RCTs, laser therapy was found to be helpful for treating melasma.

A short follow‐up period following laser therapy was one of the examined studies' weaknesses; a longer follow‐up can yield more precise information about the treatment modality's long‐term effectiveness and the likelihood of recurrence. For instance, in the Lee et al. trial, the mean MASI score decreased in the eighth week, showing a significant difference (*p* < 0.001) between Group A and Group B (868 ± 4.06 to 8.60 ± 3.88 and 8.98 ± 3.72 to 7.13 ± 2.57, respectively). However, in week 20, there was no discernible difference between the two groups' declines in the PGA, self‐assessment, and MASI score [18]. Longer follow‐up intervals can therefore measure the stability of the laser effect more precisely.

## 5 Conclusion

Out of all the RCTs that were assessed, the study from 2014 with the highest weight (6.6% weight) examined 40 patients. All of the trials, with the exception of one from 2011, had a negative mean MASI standard deviation. This study's weight was the lowest (4.2%), suggesting that the symptoms were lessened after laser therapy. At *p* < 0.00001, the final computed effect was −0.91 with a 95% confidence interval (−0.60, −1.22). Consequently, the use of laser therapy for the treatment of melasma resulted in a considerable reduction in the patients' symptoms in this meta‐analysis, which was based on the standardized versions of the MASI. Thus, another option for treating refractory melasma is laser therapy. The related recurrence, post‐inflammatory discolouration, and multiple treatment requirements of current medications restrict their effectiveness.

This treatment will continue to develop with improvements in laser equipment or technology. The MASI states that laser therapy is a melasma treatment with significant efficacy. These technologies can be advanced by imaging and drug delivery techniques, and it will be interesting to observe how fractional radiofrequency devices and picosecond lasers impact the therapy of melasma.

## Author Contributions

A.G. and M.R. designed the research study. S.M. contributed essential reagents or tools. E.A.R. analyzed the data. M.C. and A.T. wrote the paper. P.J. wrote and revised the paper.

## Ethics Statement

The study was approved by the Ethics Committee of Iran University of Medical Sciences with code: IR.IUMS.FMD.REC.1399.828.

## Conflicts of Interest

All the authors were involved in designing the study and performing the experiments. The manuscript was written and the data were analyzed with all the authors' information. All the authors read and approved the content of the article and declare no conflicts of interest.

## Data Availability

The data that support the findings of this study are available on request from the corresponding author. The data are not publicly available due to privacy or ethical restrictions.
